# The Mediating Role of Spiritual Intelligence on Well-Being and Life Satisfaction among Nurses in the Context of the COVID-19 Pandemic: A Path Analysis

**DOI:** 10.3390/bs12120515

**Published:** 2022-12-15

**Authors:** Nojoud Alrashidi, Maha Sanat Alreshidi, Wireen Leila Dator, Richard Maestrado, Sandro Villareal, Joyce Buta, Petelyne Pangket, Romeo Jr Mostoles, Analita Gonzales, Enrique Mina, Eddieson Pasay An

**Affiliations:** 1 College of Nursing, University of Hail, Hail 81491, Saudi Arabia; 2 Department of Medical-Surgical Nursing, College of Nursing, Princess Nourah Bint Abdulrahman University, Riyadh 11671, Saudi Arabia; 3 Department of Nursing, Faculty of Applied Medical Sciences, University of Tabuk, Tabuk 71491, Saudi Arabia

**Keywords:** COVID-19, spiritual intelligence, well-being, life satisfaction, nurses, Saudi Arabia

## Abstract

**Introduction:** As they are satisfied with life, nurses who demonstrate spiritual intelligence and well-being at work are tremendous assets to an organisation. This study aimed to determine the mediating effect of spiritual intelligence on the well-being and life satisfaction of nurses in the context of COVID-19. **Methods:** This research employed a cross-sectional study design. It was conducted in the Hail region of Saudi Arabia. The participants were government hospital nurses who were chosen using a multi-stage sampling method. A total of 1121 (75% response rate) nurses participated in the study. Data gathering was conducted from July to September 2022. **Results:** The life satisfaction level of the participants was deemed positive. Their well-being was better (10/15), and they rated high in spiritual intelligence (90/120). Life satisfaction had a strong association with well-being (*r* = 0.640, *p* < 0.001), but a weak association with spiritual intelligence (*r* = 0.391, *p* < 0.001). In comparison, well-being had a moderate association with spiritual intelligence (*r* = 0.551, *p* < 0.001). The direct effect of well-being on spiritual intelligence was positive and significant (β = 0.7817, *p* < 0.0001), and that of spiritual intelligence on life satisfaction was positive and significant (β = 0.1082, *p* = 0.0257). The direct effect of well-being on life satisfaction was also positive and significant (β = 1.5985, *p* < 0.0001). Conversely, well-being had an indirect impact on life satisfaction (β = 0.0846), and this effect was significant. Overall, the impact of well-being on life satisfaction was positive and significant (β = 1.6831, *p* < 0.0001). **Conclusion:** The nurses in this study were deemed satisfied, to have better well-being, and better spiritual intelligence. Life satisfaction has a strong association with well-being but a weak one with spiritual intelligence, while well-being has a moderate association with spiritual intelligence. Overall, spiritual intelligence was found to have a mediating effect on the relationship between well-being and life satisfaction. These findings suggest that an increase in spiritual growth can serve as the foundation for people to live better and more integrated lives.

## 1. Introduction

Spiritual intelligence is the ability to act wisely and compassionately while retaining inner and exterior serenity, irrespective of the circumstances [1]. It is a crucial coping technique for overcoming physical and mental health issues [2], especially during crises, such as the COVID-19 pandemic. It has a significant role in providing care during a crisis [3] and the subsequent recovery processes after a disaster [4]. Throughout the epidemic of highly contagious avian influenza in Asia, for example, it was found that spirituality was linked to increased levels of pleasant emotions, prosocial behaviour and decreased levels of lawbreaking behaviour [5]. Spiritual intelligence also played a significant role in the provision of care during the deadliest disaster of the Ebola outbreak [3]. Incontrovertibly, individuals turn frequently to spirituality or to find spiritual resources when they struggle, heal and recover during times of loss and grief [4]. Otherwise, those individuals may face mental health challenges, including difficulties with loneliness, despair and a loss of purpose in life, when their spiritual health is gravely jeopardised [6]. Promoting nurses’ spiritual intelligence can benefit their creativity, effectiveness, adaptability, communication skills and patient-centredness [7]. During the COVID-19 pandemic, it was assumed that, to provide care, nurses must have goals and meaning in their work. Consequently, spiritual intelligence could enable nurses to be able to define and control life goals and generate personal definitions, as well as to find meaning and purpose in all physical and mental events.

The definition of ‘well-being’ encompasses an individual’s assessment of the physical, social and psychological resources needed to overcome a difficulty in their physical, mental or social lives [8]. According to critics, although the clinical paradigm of well-being has drawbacks, including a lack of context sensitivity and the exclusion of results that matter to end users [9], the authors in this current study purport to examine well-being from a comprehensive perspective (i.e., depression, anxiety, energy and positive well-being). The need to consider the well-being of front-line workers has been underscored by significant evidence from comparable extreme situations, including prior epidemics [10]. Healthcare workers are subjected to a number of stressors at work, which may adversely affect their well-being [11]. Previous studies have shown the value of spiritual intelligence for an individual’s well-being [12]. Moreover, evidence points to a strong negative correlation between well-being and spiritual intelligence [13,14].

Life satisfaction is a rational method by which people evaluate the quality of their own lives in accordance with a set of hand-selected criteria [15]. It is a multi-dimensional construct representing people’s total self-assessed evaluations of their quality of life, given the importance of well-being [16]. Considering the COVID-19 outbreak, there was an indication that highly driven, productive, and efficient employees were associated with high levels of life satisfaction, which would lower their likelihood of considering leaving their jobs [17]. However, life satisfaction for nurses in a busy working environment has numerous elements that contribute to their level of life happiness, depending on their demographic characteristics, attitude and behaviour [18,19,20,21]. In fact, a significant correlation has been found between a person’s level of spiritual intelligence and how content they are with their lives [22].

The challenges that nurses have faced because of the COVID-19 pandemic have varied because they had to care for a significantly larger number of patients, and they were facing a disease regarding which there were still numerous questions and uncertainties. Furthermore, they were dealing with organisational problems at their workplace, which left them feeling fearful and uneasy [23,24]. It is assumed that during the COVID-19 pandemic, perceptions may have differed based on an individual’s experience. To the researchers’ knowledge, no previous study has been conducted to investigate the mediating effect of spiritual intelligence on well-being and life satisfaction during the COVID-19 pandemic.

By providing the capacity to create meaningful objectives and reasons for life, spiritual intelligence and well-being could also be utilised to understand the meaning of physical and mental experiences. On a daily basis, nurses face a variety of stressors in their clinical setting, which could jeopardise their well-being and cause them to lose interest in their work. In their hectic schedule, nurses pursue daily goals, both personal and professional. If, for whatever reason, they do not meet their goals, they may become dissatisfied with their lives. For nurses to respond to patients’ needs, and consider patients’ safety and organisational efficiency, it could be helpful to improve their spiritual intelligence, well-being and life satisfaction. Spiritual intelligence also aids nurses in making moral decisions. With these considerations, this study sought to determine the mediating effect of spiritual intelligence on the well-being and life satisfaction of nurses in the context of COVID-19.

## 2. Methodology

### 2.1. Design

This research was designed to be a cross-sectional study to ascertain the mediating effect of spiritual intelligence on the well-being and life satisfaction of nurses serving in public hospitals.

### 2.2. Participants/Setting

The population covered by this study were the hospital nurses assigned to 15 government-owned hospitals in the Hail region of Saudi Arabia. Nine of the hospitals were city-based and the other six were in villages. The total number of nurses directly responsible for providing direct care to patients during this study was 1690, but the actual sample size for the study was only 1121. The response rate was over 75%. The study population did not include nurse managers, nurses who declined to participate or those on leave during the survey.

Multi-stage cluster sampling was used in collecting samples from each hospital. The number of respondents from each hospital was calculated from the first sampling, which was done using Raosoft software (www.raosoft.com) (accessed on 12 October 2022), with a 5% margin of error, 95% confidence level and a response distribution of 50%. Simple random sampling was implemented for the second stage, and each prospective nurse was assigned a number upon listing their respective names. The allocated numbers were drawn using a random generator.

### 2.3. Data Collection

After clearance from the Institutional Review Board of the University of Hail and approval of the hospital authorities, the researchers contacted the key persons for coordination purposes. Nurse supervisors helped in identifying the nurses who satisfied the study criteria. They provided contact information so that the researchers established their own contact. The researchers then sent the Google Form link. The link contained information about the study and their rights as participants. Informed consent followed the information, where instructions stated that should they answer the questionnaire, then they would be providing their consent. The data were collected from July to September 2022.

### 2.4. Questionnaire

The research tool had two components. The first comprised the respondents’ sociodemographic information, including their age, gender, ethnicity, religion, level of education and number of years of hospital experience. The second contained three questionnaires that were adopted as follows: Spiritual Intelligence Self-Report Inventory [25], the Well-Being Questionnaire [26] and the Satisfaction with Life Scale (SWLS) [27].

### 2.5. Spiritual Intelligence Questionnaire

According to King [25], the questionnaire consisted of four subscales: “1. Critical Existential Thinking (CET), with seven items 1, 3, 5, 9, 13, 17 and 21; 2. Personal Meaning Production (PMP), with five items 7, 11, 15, 19 and 23; 3. Transcendental Awareness (TA) included seven items: 2, 6, 10, 14, 18, 20 and 22; and 4. Conscious State Expansion (CSE), which included five items: 4, 8, 12, 16 and 24. The nurses scored the spiritual intelligence items on a 5-point Likert scale: 0—means Not at all true of me, 1—means Not very true of me, 2—means Somewhat true of me, 3—means Very true of me and 4—means Completely true of me. All of the items were totalled, with the exception of item number 6, which was regarded as the only reverse coding.” To analyse the result, the relatively higher the score, the relatively higher the respondent’s level of spiritual intelligence [25].

#### 2.5.1. Well-Being Questionnaire

Four subscales measured the overall well-being in this questionnaire (i.e., Depression, Anxiety and Positive Well-Being with six items each, and Energy with four items). The participants scored the well-being items on a 4-point Likert scale: 0—Not at all, 1—Sometimes, 2—Most of the time, 3—All the times. Each subscale’s ratings were added after any necessary score reversals (Depression items 1, 3, 4 and 6; Anxiety items 11 and 12; Energy items 14 and 15). Higher scores on each subscale, such as those for Depression, Anxiety, Energy and Positive Well-Being, indicated more of the mood described by the subscale label. After inverting the Depression and Anxiety subscale scores, the overall score for General Well-Being was calculated by adding the subscale scores [26]. The higher the overall score, the better the well-being.

#### 2.5.2. Satisfaction with Life Scale

The respondents were asked to rate their life satisfaction on a 5-item questionnaire using the following 7-point Likert scale: Strongly disagree was expressed by 1; Disagree by 2; Slightly disagree by 3; Neither agree nor disagree by 4; Slightly agree by 5; Agree by 6; and 7 represented Strongly agree. In computing the scores and based on the interpretations for its use, they ranged from 5 to 35: a score between 5 to 9 indicated Extremely Unsatisfied; Dissatisfied from 10 to 14; Slightly Dissatisfied from 15 to 19; Slightly Satisfied between 20 and 24; Satisfied from 25 to 29; and Extremely Satisfied from 30 to 35 [27].

In this study, spiritual intelligence had an internal consistency coefficient of 0.86, well-being had one of 0.87, and life satisfaction had a Cronbach’s alpha of 0.90.

### 2.6. Ethical Consideration

This research gained clearance from the Institutional Review Board of the University of XXX. The participants’ rights, privacy, anonymity and confidentiality were thoroughly ensured.

### 2.7. Statistical Analysis

The demographic information was calculated into frequency and percentage numbers. To determine the causality of the relationship of spiritual intelligence, well-being and life satisfaction, the correlation, multiple regression (path analysis) and the Statistical Package for the Social Sciences, version 26 (SPSS v.26), were used to conduct the statistical analyses; however, for the path analysis modelling tool, the PROCESS macro was used. The significance level was set at 0.05 for each value.

## 3. Results

A total of 1121 nurses were part of the study. These nurses were primarily female (96.3%), 30 years old and above (67.2%), non-Saudi nationals (52.0%), Muslims (58.1%), with bachelor’s degrees (87.5%) and with more than 10 years of hospital experience (68.8%) (Table 1).

Table 2 presents the level of life satisfaction, well-being and spiritual intelligence of the participants. During the pandemic, the participants perceived their life satisfaction as “slightly satisfied” but a better well-being, while they perceived that they have good spiritual intelligence.

Table 3 presents the association between well-being, life satisfaction and spiritual intelligence. Life satisfaction has a strong association with well-being (*r* = 0.640, *p* < 0.001), but a weak association with spiritual intelligence (*r* = 0.391, *p* < 0.001, and well-being has a moderate association with spiritual intelligence (*r* = 0.551, *p* < 0.001)

Table 4 presents PROCESS model type 4. The models were found to be significant, with R values ranging from 0.5514 (*p* < 0.0001) and 0.6557 (*p* < 0.0001). The well-being elucidates 30.41% of the change in spiritual intelligence (First Model), and well-being and spiritual intelligence explain 42.99% of the change in life satisfaction (Second Model).

Table 5 shows the direct and indirect effects of the mediation analysis.

### 3.1. Direct Effects

**Figure 1:**
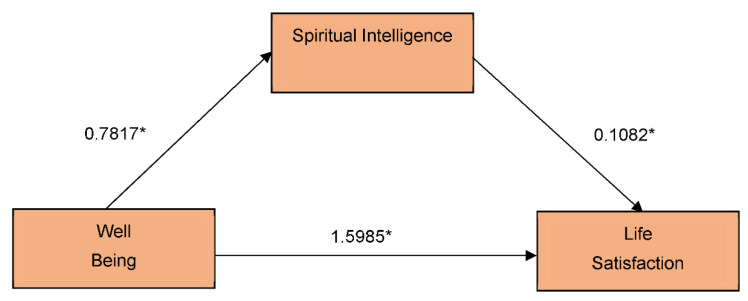
Direct effects on the postulated model; * significant at 0.01.

The first section of the table displays the impact of well-being on spiritual intelligence. There is a positive and considerable direct effect of well-being on spiritual intelligence (β = 0.7817, *p* < 0.0001). The direct result of spiritual intelligence on life satisfaction is positive and significant (β = 0.1082, *p* = 0.0257). In addition, the direct effect of well-being on life satisfaction is positive and significant (β = 1.5985, *p* < 0.0001) (see Figure 1) Since this coefficient is the primary mediator predictor, it is important to investigate both the direct and cumulative effects of well-being on life satisfaction.

### 3.2. Indirect Effects

Well-being has an indirect impact on life satisfaction (β = 0.0846), and this effect is significant (bootstrapped 95% confidence interval LLCI = 0.0270 and ULCI = 0.1439, which does not include zero). Finally, the overall impact of well-being on life satisfaction is positive and significant (β = 1.6831, *p* < 0.0001).

## 4. Discussion

In this study, the level of life satisfaction was deemed “slightly satisfied”, which implies that the nurses had a normal equilibrium between their present state and their individual goals. According to recent research, nurses are generally satisfied with their lives [21,28]; however, some aspects of their lives could be improved [29]. Nurses who are satisfied with their jobs are more efficient and feel more devoted to their jobs [30]. It has also been shown that nurses’ job satisfaction influences their satisfaction with the services provided [31]. The well-being of the nurses seemed better; this indicates that nurses are aware of an individual’s overall, psychological, interpersonal, and environmental state, each of which interacts with the others and has a different level of significance and influence. In several studies, the authors indicated that high degrees of psychological anguish are prevalent among Saudi Arabian nurses, which threatens their well-being [32,33,34].

The spiritual intelligence of the participants was high, implying that the nurses have an intrinsic ability to comprehend existential issues, life’s purpose and the seamlessness of a person’s connectedness to the rest of the world. This current finding coincides with the study of Yadollahpour et al. [35], which specified that nurses benefit in various ways from the encouragement of spirituality at work, including developing a favourable opinion of the company and growing their devotion to it, adhering to work more diligently and thus experiencing more job happiness. The purpose of spirituality is to increase people’s awareness and spread the principles that make life and work better for nurses [36]. The hardships of daily living are merged with one’s spirituality in the community and profession. Nurses who perceive a spiritual connection to their profession display inner strength, tranquillity, patience at work, calmness and a good outlook [35]. Consequently, nurses’ spirituality helps them to cope with complex conditions, especially at work [37]. In the Alquwez et al. [38] study, during a crisis, the spiritual well-being of nurses was found to be just as important as the other aspects of their lives. The bottom line is that nurses with high spirituality have resilience and inner strength., which increases their capacity for adapting to threatening situations more skilfully, handle stressful situations, get through conflict, and adjust to adversity. Additionally, they are as regards better psychological health, which may aid their ability to cope with work-related stress [35,39]. These results contribute to hospital administrators, so much so that nursing staff should have their well-being and spiritual intelligence frequently checked by the administration. Additionally, a lower tier of life satisfaction, well-being, and spiritual intelligence correlates with a higher desire to quit one’s job and, as a result, a staff shortage. The amount of nursing care allocated is strongly impacted by inadequate personnel resources [40].

Life satisfaction has a strong association with nurses’ well-being, which means that they enjoy their lives while living well, being capable of acting independently and autonomously, feeling good about themselves and having a purpose in life. This finding advocates those of similar studies [41,42]. However, Lorber et al. [28] noted that nurses working in Slovenian hospitals reported modest levels of life satisfaction and well-being. The health of nurses was also shown to be moderate in other studies [43,44]. It is possible to explain disparities in nurses’ happiness and well-being from hospital to hospital using the results of the earlier study, which emphasised variations in organisational culture [45], organisational support [46], and leadership style [47]. Management staff must understand the value of employee well-being to the organisation, as it fosters improved health, positive self-esteem, stronger interpersonal relationships and resilience [28]. In contrast, life satisfaction has a weak association with spiritual intelligence, which implies that nurses who generally consider their life to be successful likely lack the abilities and behaviour capabilities required to apply spirituality. This finding challenges other research [48,49,50] that found that spiritual intelligence and its components enable people to think deeply about the spiritual considerations, use resources to address difficulties in their lives and act morally [51], all of which boost life satisfaction. Spiritual intelligence fosters internal harmony and enables nurses to appreciate the enjoyable side of life [48]. A study in Iran similarly indicated that increased spiritual intelligence resulted in increased life satisfaction [52]. Nurses with high spiritual intelligence ratings are more aware and employ more resources for problem-solving. They have a more optimistic outlook on the world and are more content in their lives because they have greater ethical qualities [52]. The level of spiritual intelligence can make a significant difference in many areas of life, most notably in life satisfaction. Through this, hospital management staff can ascertain the actual scenario and attempt to understand how crucial it is to monitor workers’ life satisfaction, well-being and spiritual intelligence both to improve employee health and to produce better results. The practical ramifications of the findings imply that treatments aimed at fostering positive life satisfaction, well-being and spiritual intelligence may contribute to improving nursing environments, which may lead to improved methods for ensuring safety and quality as well as improved nursing outcomes.

Well-being has a moderate association with spiritual intelligence, which means that nurses’ well-being can be influenced by their spirituality and vice versa by the actions that they take to serve others and practice compassion. This finding validates those of other studies [53,54,55]. Spiritual and religious beliefs provide nurses with hope to consider having a peaceful life by enabling them to understand a few psychological strains placed on them and undesired occurrences that positively impact them [55]. They are happier and more fulfilled physically and spiritually, which makes them more likely to be compassionate towards other people and their patients [56]. Believing in a higher power and a higher goal leads to happiness [57]. Nurses have optimistic thoughts and behaviours, see things in a positive light and work hard to achieve success [58]. Some studies conclude that spirituality’s influence on health significantly affects how long people live and how likely they are to contract diseases [59]. Promoting nurses’ spiritual intelligence can support their efforts to live more holistically, recognize different life patterns, improve communication skills, have a professional sense of a larger objective, understand the true significance of events, create meaningful work environments, and be happier in a more sustainable way. Nurses who are happy in their work environment have a sense of well-being. They can benefit not only themselves but also their co-workers and the organisations for which they work, because they are more productive, capable of making wiser choices, and have better interpersonal interactions [60].

The impact of well-being on spiritual intelligence is positive and significant, which means that it is possible to hypothesise that those with high spiritual intelligence can reduce stress because they adhere to a higher power and practice a religion, which is more prevalent than other psychological or physical crises, and presents opportunities to learn and develop. Spiritual nurses exhibit virtues such as kindness, compassion, empathy, forgiveness, and charity [61]. It follows that nurses with high spiritual intelligence have better stress management skills since spiritual intelligence has a favourable and large direct effect on life satisfaction. Compared to other professions, nurses are more likely to experience life crises and learn from them. Spiritually intelligent nurses display qualities such as humility, kindness, humanism, compassion, and love [62]. In addition, nurses with high spiritual intelligence are stabler, more confident in themselves and better able to handle problems at work [62]. Accordingly, barriers are eventually eliminated while enhancing individual effectiveness and performance, workplace communication and mutual understanding [63]. Workplace spiritual intelligence can infuse organisations with humanity to foster responsibility and productive environments, laying the groundwork for successful operations and, ultimately, satisfaction with life. Such a result of the present study agrees with several other studies [63,64]. They contend that utilising spiritual intelligence can help to solve issues based on their importance and status and that it also allows individuals to be aware of how well they are performing in achieving their objectives and how much satisfaction they are receiving because of their actions.

Since well-being has a positive and large direct effect on life satisfaction, it is likely that well-being in all of its dimensions—mental, physical, and social—is what enables one to keep a successful and upbeat attitude on life and, therefore, is a source of life satisfaction. According to Hashemi and Abbasi [63], striving for perfection in a person’s realisation of their true potential constitutes well-being. From this perspective, welfare refers to an effort to advance and transcend that shows up in the realisation of a person’s skills and talents. Undoubtedly, adopting this behavioural strategy can contribute to life satisfaction. Given that nurses’ physical and mental health are prone to risk, it could be a crucial factor in determining how satisfied they are with their lives. Because they create the groundwork for increasing the spiritual intelligence of nurses and other healthcare professionals, it is suggested that this association be maintained, as this study showed a favourable and significant link between well-being and life satisfaction. In hospitals and health facilities, a more meaningful approach to well-being and its components has been adopted, considering the broader context of expanding religious beliefs.

The mediation analysis’s findings demonstrated that well-being has a strong indirect impact on life satisfaction. Finally, the overall impact of well-being on satisfaction with life is positive and significant, which suggests that the nurses lived well, and could include making possible a satisfying life experience that has been fully lived. Tekir’s [65] study reported that being in a state of well-being involves having an optimistic personal view, being aware of personal strengths and weaknesses, accepting oneself for who one is, being content with oneself, being able to act on one’s initiative and finding meaning in one’s life. As research advocates [65,66,67], well-being and life satisfaction are closely related. The degree to which a person contributes to society is their level of well-being. To handle and adjust to difficult events, such as the important cases they come across in their professional setting, nurses need a high level of well-being. Being in good health means having the inner strength to act on your resolve and accept your own experiences [68]. Adopting this behaviour can certainly enhance life satisfaction. Life satisfaction is essential in nurses’ lives because it helps them cope with stress and achieve their goals [65].

This study has implications for nursing practice because the ability to live without worry, fear or anxiety facilitates an increase in spiritual growth, making nurses stronger and creating opportunities for them to participate in novel activities and carry out their duties with greater clarity, meaning and purpose. Because fear of change comes from one’s mind and not from the environment around them, spiritual intelligence aids people in overcoming this fear. Growth in one’s capacity to understand emotions and assist others in better emotional control results from developing spiritual intelligence. The development of spiritual intelligence skills at all levels and for all people has a significant impact on the fulfilment of transcendentalism and the significance of society.

### Study Limitations

A few limitations apply to this study. However, such limitations can be addressed by further inquiry, by taking the aforementioned limitations and using qualitative design to widen the perspective regarding the mediating effect of spirituality on well-being and life satisfaction. The exclusion of private hospitals, nurses with managerial positions and failure to correlate other demographics (e.g., nationality, marital status) can all be cited as limitations. Primary healthcare nurses may also participate in the study to have their perspectives included.

## 5. Conclusions

The nurses in this study were deemed satisfied and to have better well-being and spiritual intelligence. Although life satisfaction had a strong association with well-being, it had a weak one with spiritual intelligence, while well-being had a moderate association with spiritual intelligence. Overall, spiritual intelligence was found to have a mediating effect on the relationship between well-being and life satisfaction. These findings suggest that an increase in spiritual growth can serve as the foundation for people to live better and more integrated lives.

## Figures and Tables

**Table 1:** Demographic profile of the participants. N = 1121.

Characteristics		Frequency	Percent
Age of Nurse	Below 30 y.o.	368	32.8
	30 y.o. and above	753	67.2
Sex	Male	41	3.7
	Female	1080	96.3
Ethnicity	Saudi	538	48.0
	Non-Saudi	583	52.0
Religious Affiliation	Muslim	651	58.1
	Christian	357	31.8
	Others	113	10.1
Educational Level	Diploma	0	0.0
	Bachelor	981	87.5
	Master’s	140	12.5
Years of Hospital Experience	Below 10 years	771	68.8
	10 years and above	350	31.2

**Table 2:** Descriptive statistics—path variables.

Characteristics	Range	Mean ± SD
Life Satisfaction	2.60–7.00	5.75 ± 0.936
Well-being	1.14–3.00	1.71 ± 0.367
Spiritual Intelligence	2.08–4.00	2.89 ± 0.523

**Table 3:** Association between well-being, life satisfaction, and spiritual intelligence.

Variable	1	2	3
1. LS	1		
2. WB	0.640 **	1	
3. SI	0.391 **	0.551 **	1

***Note***: LS—Life Satisfaction, WB—Well-being, SI—Spiritual Intelligence; Level of significance: * *p* < 0.05, ** *p* < 0.01.

**Table 4:** Regression analysis on well-being.

Outcome	R	R^2^	MSE	F	df1	df2	*p*-Value
SI	0.5514	0.3041	0.1910	244.2347	2.0	1118.0	<0.0001
LS	0.6557	0.4299	0.5013	280.8047	3.0	1117.0	<0.0001

***Note***: LS—Life Satisfaction, WB—Well Being, SI—Spiritual Intelligence; Level of significance: * *p* < 0.05, ** *p* < 0.01.

**Table 5:** Mediation analysis on life satisfaction.

Direct Effects on SI	Coefficient	SE	t-Value	*p*-Value	LLCI	ULCI
WB	0.7817	0.0360	21.7177	<0.0001	0.7111	0.8523
Direct effects on LS	Coefficient	SE	t-value	*p*-value	LLCI	ULCI
WB	1.5985	0.0695	22.9876	<0.0001	1.4621	1.7349
SI	0.1082	0.0485	2.2336	0.0257	0.0132	0.2033
Indirect effects on LS	Effect	BootSE	Significance		BootLLCI	BootULCI
WB → LS	0.0846	0.0297	Significant		0.0270	0.1439
Total effect WB on LS	Effect	SE	t-value	*p*-value	LLCI	ULCI
Total effect	1.6831	0.0584	28.8104	<0.0001	1.5685	1.7977

***Note***: LS—Life Satisfaction, WB—Well Being, SI—Spiritual Intelligence; Level of significance: * *p* < 0.05, ** *p* < 0.01.

## Data Availability

The data presented in this study are available on request from the corresponding author.
